# Identification and Interpretation of A-to-I RNA Editing Events in Insect Transcriptomes

**DOI:** 10.3390/ijms242417126

**Published:** 2023-12-05

**Authors:** Ye Xu, Jiyao Liu, Tianyou Zhao, Fan Song, Li Tian, Wanzhi Cai, Hu Li, Yuange Duan

**Affiliations:** MOA Key Lab of Pest Monitoring and Green Management, Department of Entomology, College of Plant Protection, China Agricultural University, Beijing 100193, China; xuye@cau.edu.cn (Y.X.); liujiyao@cau.edu.cn (J.L.); tianyou96@outlook.com (T.Z.); fansong@cau.edu.cn (F.S.); ltian@cau.edu.cn (L.T.); caiwz@cau.edu.cn (W.C.); tigerleecau@hotmail.com (H.L.)

**Keywords:** A-to-I RNA editing, identification, methodology

## Abstract

Adenosine-to-inosine (A-to-I) RNA editing is the most prevalent RNA modification in the nervous systems of metazoans. To study the biological significance of RNA editing, we first have to accurately identify these editing events from the transcriptome. The genome-wide identification of RNA editing sites remains a challenging task. In this review, we will first introduce the occurrence, regulation, and importance of A-to-I RNA editing and then describe the established bioinformatic procedures and difficulties in the accurate identification of these sit esespecially in small sized non-model insects. In brief, (1) to obtain an accurate profile of RNA editing sites, a transcriptome coupled with the DNA resequencing of a matched sample is favorable; (2) the single-cell sequencing technique is ready to be applied to RNA editing studies, but there are a few limitations to overcome; (3) during mapping and variant calling steps, various issues, like mapping and base quality, soft-clipping, and the positions of mismatches on reads, should be carefully considered; (4) Sanger sequencing of both RNA and the matched DNA is the best verification of RNA editing sites, but other auxiliary evidence, like the nonsynonymous-to-synonymous ratio or the linkage information, is also helpful for judging the reliability of editing sites. We have systematically reviewed the understanding of the biological significance of RNA editing and summarized the methodology for identifying such editing events. We also raised several promising aspects and challenges in this field. With insightful perspectives on both scientific and technical issues, our review will benefit the researchers in the broader RNA editing community.

## 1. Introduction

**Figure 1:**
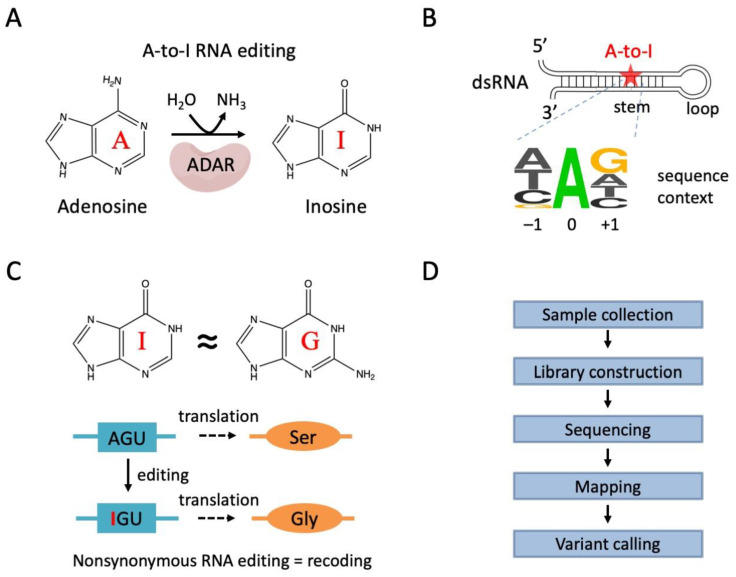
A basic introduction of A-to-I RNA editing in metazoans. (**A**) A-to-I RNA editing is a deamination reaction mediated by ADAR enzymes. (**B**) Cis-elements preferred by ADAR: double-stranded RNA and a 3-mer motif favoring an upstream non-G and a downstream G. (**C**) Inosine is recognized as guanosine. A-to-I editing resembles A-to-G mutation. Editing sites in the CDS might cause nonsynonymous mutations, recoding the genomic information. (**D**) Sample collection and the subsequent traditional pipelines for the identification of A-to-I RNA editing sites from the transcriptome.

Adenosine-to-inosine (A-to-I) RNA editing takes place in the neuronal transcriptomes of various metazoans [1], ranging from corals [2], worms [3], insects [4,5], mollusks [6,7], to vertebrates [8,9,10,11]. The enzyme, named adenosine deaminase, acting on RNA (ADAR) [12] converts adenosines to inosines in RNAs (Figure 1A). A-to-I editing is prevalent in the RNA pool, but the editing events are not random, and not all adenosines in the transcripts are “editable”. Specifically, ADAR favors the adenosines in the double-stranded RNA (dsRNA) structure within a particular sequence context (Figure 1B). Although the strong target preference of ADAR excludes many adenosines from being edited, there are still millions of editable adenosines in the transcriptomes of different species. For example, it is estimated that over one hundred million adenosines in the human genome are potentially editable [13], making A-to-I editing the most prevalent RNA modification in animals. Intriguingly, inosine is recognized as guanosine by cellular machineries, and thus A-to-I RNA editing has similar consequences to A-to-G DNA mutations [14]. Extensive editing would dramatically re-write the transcriptome beyond the genome sequence [15]. Particularly, editing events in the coding sequence (CDS) might alter the amino acid and recode the genome (Figure 1C). As a consequence, nonsynonymous RNA editing events are also termed “recoding” events [6]. Studies in large animals like cephalopods have revealed that extensive nonsynonymous editing in neuronal transcripts would strongly affect the protein function and facilitate organisms adapt to a capricious environment [7,16,17,18].

## 2. A-to-I RNA Editing in Different Animal Clades and the Functional Innovation

Although human RNA editing sites are highly abundant across the transcriptome, most sites are located in *Alu* repetitive elements [19,20]. These editing sites collectively prevent MDA5 from sensing endogenous dsRNA as “non-self” [21]. In such cases, the importance of each individual editing site is weakened. The immune-protector role is achieved via the collective effect of numerous editing sites, and each site only has a very low expression level and editing level. Identification of new editing sites in repetitive elements does not deepen our understanding of the function of RNA editing because all these editing sites have the same task. This phenomenon, where the repetitive editing sites are highly abundant and act as an immune-protector, is conserved in mammals [11,14,22,23,24,25]. At the class level, the other animal classes with systematic RNA editing studies in multiple species are the insect class (Insecta) and the cephalopods (Cephalopoda, including octopus, squid, and cuttlefish).

In sharp contrast, the composition and distribution of RNA editomes in insects and cephalopods are completely different from what is known for the mammalian species. Insects only have one *Adar* gene, which is orthologous to *ADAR2* among the three mammalian *ADAR*s [26,27,28]. Apart from the catalytically inactive ADAR3, the mammalian ADAR1 and ADAR2 have some overlapped target regions when both enzymes are co-expressed [23,29,30], but the two ADAR paralogs still have distinct functional divergence where ADAR1 mainly targets non-coding repeats and ADAR2 mainly targets the exonic region of RNA [12,31]. The homology between insect Adar and mammalian ADAR2 dictates that the majority of “regular” RNA editing sites in insects take place in the exonic region or CDS of neuronal genes, diversifying the neuronal proteome. Here, the regular editing sites are conceptually opposite to the hyper-editing sites described in the following section. Although the abundance of insect RNA editing sites is not comparable to the rampant editing in human *Alu* [19,20], each recoding site in insects has its unique function to the host genes that might affect the organism in many different ways. The numerous combinations of different recoding sites would exponentially increase the proteomic complexity in neurons. The same conclusion of the proteomic diversifying role of nonsynonymous RNA editing has been proposed in cephalopods [7,32]. Although cephalopods have both ADAR1 and ADAR2, the majority of RNA editing sites are located in the CDS and cause nonsynonymous changes, diversifying the neuronal proteome in a spatiotemporal manner [7,16,17,18]. Therefore, the various recoding editing sites in insects and cephalopods are highly informative. Virtually every single newly discovered recoding site is valuable to the research community and might add novel knowledge to our current understanding of RNA editing.

Comprehensive identification of RNA editing sites in various species of great importance. The conserved editing sites across species may have great significance, and the species-specific RNA editing may have unique significance in that species. Whether we aim to study the conserved or species-specific editing sites or even the within-species variation in editing sites, the first step is to accurately profile the RNA editome in each species/strain. Without the comprehensive identification of RNA editing sites in various organisms, the conservation analyses could not be performed. Our notion here is widely supported by studies on the conserved editing sites in mammals [33,34], the conserved and species-specific editing sites in *Drosophila* [35,36], the conserved and species-specific editing sites in cephalopods [7,32], and the variation in RNA editing at population level in flies and humans [37,38].

The importance of discovering each single editing site complicates the accurate identification of A-to-I RNA editing. Traditionally, this process requires five steps: (1) sample collection; (2) library construction; (3) sequencing; (4) mapping; and (5) variant calling (Figure 1D). In the following sections, we will introduce the basic concept, methodology, and challenges/guidance within each step.

## 3. Limitations in Studying RNA Editing: RNA-Seq and the Matched DNA-Seq Should Be Obtained

Sample collection is the prerequisite for many kinds of studies. Compared to the rapidly emerging studies on RNA editing in large animals like mammals [30,31,39,40,41] and cephalopods [6,7,16,17,32], the genome-wide A-to-I RNA editomes in insects were only studied for a few representative species (clades) like *Drosophila* [35,36,42,43], bees [5], ants [4], and moths [44]. Insecta is the largest class in the animal kingdom, but the few studies covering species with genome-wide RNA editing only cover Diptera, Hymenoptera, and Lepidoptera, leaving the largest order, Coleoptera, unexplored. The number of RNA editing studies in insects does not match the great biodiversity in this clade. We will discuss the cause of this disbalance and stress that the sample collection and library preparation/construction processes are crucial steps that determine the feasibility of studying RNA editing.

**Figure 2:**
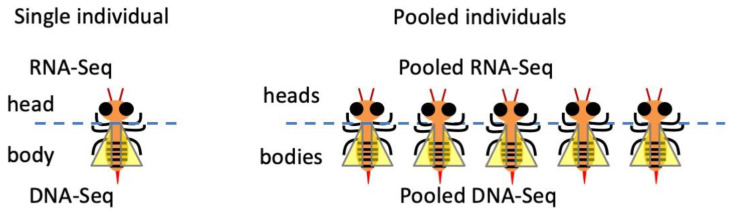
Preliminary steps for RNA editing detection. Sample preparation for identifying A-to-I RNA editing sites. RNA from heads and DNA from matched individuals should be sequenced.

To fully explain the difficulty in RNA editing studies, we should first clarify (disambiguate) the term “RNA editing identification/detection”. This term not only refers to determining the location or number of editing sites but also the quantification of RNA editing level, which is the fraction of edited RNA molecules among total RNA molecules. Thus, the several experimental strategies to enrich the inosine-containing RNAs [45,46] might not be suitable for quantifying editing level because the unedited RNAs are largely missing. While acknowledging the contribution of the “inosine enrichment” approaches to the finding of editing sites, in this review, we will only discuss the library construction strategies that faithfully capture all mRNAs in the cellular system because quantification of editing levels for different sites would be indispensable for the evolutionary analyses on nonsynonymous and synonymous editing sites [7,33,47,48,49]. The transcriptome-wide detection of RNA editing events in a species requires (1) the head/brain transcriptome of a single individual; (2) ideally, the matched DNA resequencing of the same individual [4,7] (usually the body or leg is sequenced; Figure 2). Furthermore, (3) if the sequencing of head RNA from a single individual is not applicable, then the heads from pooled individuals should come from inbreeding lines or isogenic lines to exclude confounding factors like single-nucleotide polymorphism (SNP), or the DNA from pooled bodies should be re-sequenced to match the pooled heads (Figure 2) [4]; (4) if the above requirements for DNA data are not applicable then the species must have a well-annotated SNP database, like the 1000-genome project in human or *Drosophila melanogaster* [50,51,52], to remove the potential false-positive sites during RNA editing detection. Briefly, criteria (1) and (2) are the standard protocol guiding the sample preparation for RNA editing studies, and criteria (3) or (4) are the backup strategies when (2) is not available.

Large animals like mammals and cephalopods are well suited for criteria (1) and (2) of RNA editing identification, and this judgement agrees with the fact that various species in these clades have systematic studies on the transcriptome-wide RNA editing profile [6,7,11,14,16,17,22,23,24,25,32]. However, most insect species do not meet these criteria for RNA editing identification. Criterion (4) exclusively refers to model organism *Drosophila melanogaster*. For criterion (3), the inbreeding line is also very rare for non-model insects. For criteria (1) and (2), many insects are too small to extract sufficient RNA from a single head. Low RNA concentration will lead to the failure of library construction. For the pooled strategy, it only applies to a few insects that could be raised in laboratories because it is difficult to collect sufficient numbers of individuals for most wild species. Therefore, the limiting steps of studying RNA editing in insects lie in the sample collection and library construction. Apart from studying model animals raised in labs, which have clear genomic backgrounds, there is also an undoubted importance for studying the genetics and genomics of wild animals in order to understand the cis-regulatory elements as well as the connection between genotype and phenotype, such as the field termed population genetics/genomics. Specifically, the RNA editing studies in wild animals are restricted by the several obstacles mentioned above.

In model insect *D. melanogaster*, there are many inbreeding or isogenic lines (strains), such as the OregonR, *w^1118^*, and ISO1. The same strain has an identical genetic background, and no individual-specific SNPs are present to disrupt the identification of RNA editing sites. With this convenience, multiple individuals could be pooled to sequence the head RNA and body DNA (Figure 2). Alternatively, if the reference genome of the particular fly strain is available then it is unnecessary to re-sequence the DNA of the matched individual. Due to its well-known background, *D. melanogaster* is not a typical example representing the situation in common insects. Our conclusion is that an RNA-Seq dataset coupled with matched DNA-Seq is still quixotic for most (not all) insect species. In contrast, this is not a problem for large non-model animals like cephalopods (octopus, squid, and cuttlefish) [6,7,32].

## 4. Detecting RNA Editing in Single Cells Is Promising but Challenging

Notably, with recent advances in the single-cell RNA-sequencing (scRNA-Seq) technique, one may expect that the sample collection and acquisition of matched RNA + DNA for small animals (like insects) should be relatively simple, as modern libraries can be constructed at the single-cell level. However, this approach is not yet widely applied to many animal species, including insects, but efforts have been made to find RNA editing events from scRNA-Seq. Here, we (1) first theoretically introduce the concept that insect cells are not suitable for single-cell separation compared to mammalian cells; (2) we present a data and literature search to show that the existing scRNA-Seq data for insects are indeed extremely rare compared to the plethora of scRNA-Seq data in mammals, presumably due to the technical limitations in obtaining single cells from insects; (3) introduce the fact that the currently popular scRNA-Seq strategy only sequences the 3′-end of mRNA, and this approach is not suitable for application to RNA editing studies; (4) finally, we anticipate that the scRNA-Seq strategy that covers the full-length mRNA is useful for RNA editing detection, although it still suffers from detection bias due to limited data size per cell. Our point is that studying RNA editing at the single-cell level will gradually become the trend in the RNA editing community.

Single-cell RNA sequencing libraries require the separation of single cells followed by library construction. This cell separation step was mainly designed and optimized for mammalian cells [53]. The application of this experimental approach to other animal clades faces strong challenges. Take insects for instance; while the original scRNA-Seq technique appeared in 2009 [53], its application to insects was only achieved very recently [54]. The reason is that many insect cells from adults are encapsulated by the exoskeleton, which prevents the cells from being separated intactly (explained in [54]). The exoskeleton might not be an issue for larva, but there is always the need to understand the single-cell profile for adult insects. Researchers could only manage to find a way to isolate the nuclei of insect tissues and then perform the traditional library construction and sequencing steps [54]. Although this methodology is promising for any other animal species, the fact is that the scRNA-Seq data remain very rare for insects compared to mammals, let alone studying RNA editing using the single cell data.

We found a few studies of RNA editing using scRNA-Seq [55,56,57,58], including three papers on humans [55,56,57] and one paper on mice [58], while no insect species were investigated. From these facts, we see a promising trend that researchers are trying to identify RNA editing events from the single-cell data, but the scarcity of relevant studies might reflect some unresolved technical limitations behind this idea, especially for non-model insects. Moreover, we should also consider an issue related to the funding provided and the research cost/benefit ratio. The health investment strongly influences human research, while the research in non-mammalian organisms focuses on other considerations. The investment is unequal for all the organisms. We believe that the lack of funding is another reason for the poor representation of data in non-mammalian organisms. Although the exoskeleton can be a limitation for insect cell isolation, there might be ways to overcome this limitation when sufficient funding is provided. For insects, the effort to optimize a method might not be rewarding in the present scientific environment.

Here, we carry out an interesting temporal comparison. Soon after the invention of next-generation sequencing (NGS) on whole transcriptome (bulk RNA-Seq, Illumina, San Diego, CA, USA) in 2007, the bioinformatic pipeline(s) for systematic identification of A-to-I RNA editing events and the corresponding online databases rapidly emerged in 2010 [59,60,61,62,63,64]. In sharp contrast, although the scRNA-Seq technique was invented in 2009 [53], the use of scRNA-Seq data to identify RNA editing sites first appeared in 2016 [56]. Given the highly mature bioinformatic pipelines/tools for analyzing RNA editing in bulk RNA-Seq [65,66,67,68], detection of RNA editing events in scRNA-Seq data should have been elucidated sooner. This temporal gap between the emergence of scRNA-Seq and its usage in RNA editing suggests that there might be hidden obstacles/limitations in the practice of these pipelines, such as the aforementioned issues for non-model organisms and the detection bias we introduce in the following.

ScRNA-Seq libraries have two major types. The strategy of one type is to sequence the fragments from full-length mRNAs [53], and the other strategy, with an example being Drop-Seq, typically sequences the 3′-end of mRNAs [69]. The major purpose of scRNA-Seq is to obtain the gene expression profile of a cell. Given the plethora of cells to be sequenced, the data size per cell is limited so that the sampling bias of sequencing reads is the main confounding factor that leads to the inaccurate quantification of gene expression. Compared to sequencing the fragments from full-length mRNA, only sequencing the 3′-end saves time and effort of obtaining reads from more genes, increasing the accuracy and reducing the variance of gene expression profile. To perform gene expression analyses at the single-cell level, the 3′-end strategy is a more popular choice than covering the whole mRNA. Undoubtedly, this smart strategy has greatly facilitated the broad community of cancer research [70]. In contrast, for RNA editing analysis, a basic requirement is to obtain the RNA coverage in an unbiased way. The popular 3′-end strategy of scRNA-Seq does not fit for RNA editing studies. Thus, one arrives at the full-length mRNA strategy, but this strategy still has its limitations. Conceivably, compared to the quantification of gene expression, the accurate identification of RNA editing sites is more sensitive to sequencing coverage. As mentioned above, the full-length mRNA strategy of scRNA-Seq suffers from limited reads per cell, jeopardizing the precise detection of RNA editing events. Nevertheless, bioinformatics aims to fully take advantage of existing data, and the full-length scRNA-Seq is already the best approach to help study RNA editing at the single-cell level [55,56,57,58]. Moreover, bulk RNA-Seq data accompanying the scRNA-Seq data are highly preferred. Thus, we anticipate this idea to be spread to more species in the future.

In this part, we first present theoretical evidence that insect cells are not favorable for constructing scRNA-Seq data and then provide statistical data to show that scRNA-Seq for insects is indeed very rare. We found that both strategies of scRNA-Seq inevitably have shortcomings in RNA editing detection, but bioinformaticians have devoted efforts to achieving this goal [55,56,57,58], and we can anticipate the broad application of RNA editing ideas to the scRNA-Seq data in the near future.

## 5. The Importance of Mapping: Attempts with Different Aligners

**Figure 3:**
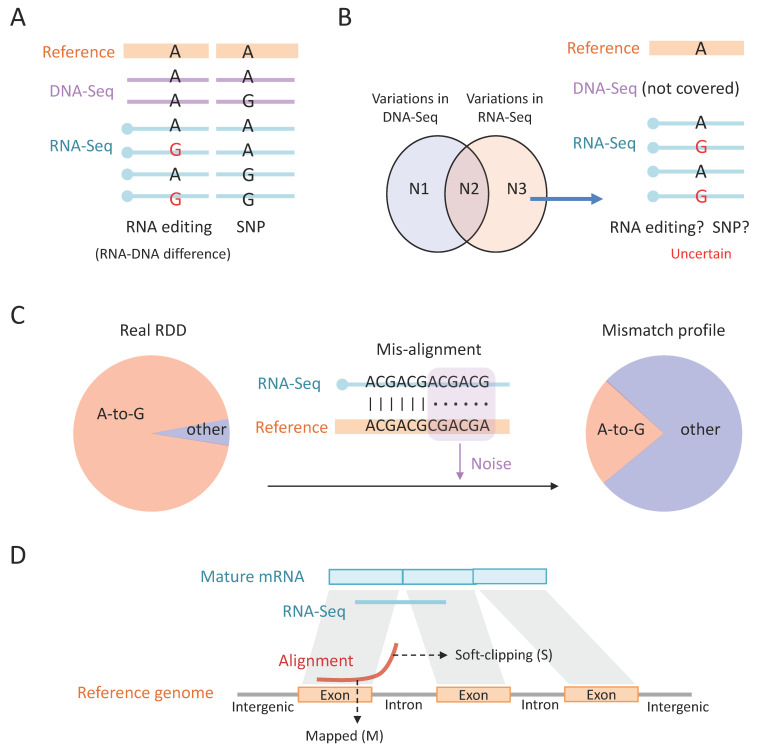
Diagram illustrating how to find RNA editing sites from sequencing data. (**A**) RNA editing is found by looking for RNA–DNA difference (RDD). SNPs could be excluded by sequencing the DNA from matched individuals. (**B**) Even with DNA-Seq data, SNPs could also “hide” in the regions where DNA reads are not covered. Then, the RNA editing sites might also be false-positive. (**C**) Mismatches introduced by misalignments are artefacts which will dilute the real A-to-I (G) RNA editing signal. (**D**) Soft-clipping of RNA-Seq reads usually occurs at splicing junctions for the part of the read mapped to the reference genome. Soft-clipping is also a source of undesired false-positive mismatches.

With RNA-Seq and DNA-Seq data in hand, the next step is to map the sequencing reads to the reference genome. To identify the A-to-I RNA editing sites, one should map the RNA-Seq and DNA-Seq to the reference genome and look for the positions where the DNA reads support the reference genome and the RNA reads show variations (Figure 3A). This strategy aims to find the real RNA–DNA difference (RDD), which could only be explained by RNA editing. Ideally, over 90% of the RDDs are A > G variations, representing A-to-I RNA editing [4,7,66]. This demonstrates the necessity of using both DNA-Seq and RNA-Seq. Alternatively, without a matched DNA-Seq, the difference between RNA-Seq and the reference genome could come from SNPs (Figure 3A). Notably, although it is well known that SNP sites should be discarded in the RNA editing studies, the method of excluding SNPs is sometimes misused. For example, it is a logical flaw to think that the variations in RNA-Seq minus the variations in DNA-Seq equal RNA editing sites (Figure 3B). While the variations in RNA-Seq or DNA-Seq are obtained by mapping the reads to the reference genome, respectively, the above-mentioned logic ignores the situation where a region has no DNA-Seq covered and the variations in RNA-Seq actually reflect the potential SNPs (Figure 3B). Thus, finding the real RDD should require sufficient DNA coverage with no alternative alleles at these positions.

The above-mentioned analyses to look for RDD all rely on the reads being accurately aligned. Indeed, mapping the sequencing reads to the reference genome is commonly used in bioinformatic works. The importance of this mapping step is usually underestimated. For most bioinformatic studies involving transcriptome (RNA-Seq) data, the only purpose of using RNA-Seq is to calculate a relative expression level of genes or perform differential expression analysis. Such analyses do not require highly accurate mapping of reads because the misalignments of a few reads would not skew the global differential expression patterns [48]. In sharp contrast, for the other uses of RNA-Seq data that involve the mismatch information or to detect very slight changes in expression or splicing, the accuracy of mapping would strongly affect the result.

Conceivably, misalignments will introduce undesired mismatches (Figure 3C). These mismatches are artefacts that do not reflect the real mutations in the sequence. The artefacts are random and will severely dilute the fraction of true positive A-to-G mismatches (Figure 3C). Among the total reads in an RNA-Seq library (>10^7^), only a small fraction contains regular A-to-I editing events (e.g., <1%) [43,48], suggesting that a few misaligned reads will produce excessive noise to confound the mismatches profile (Figure 3C). To reduce the misalignments, a feasible approach is to align the reads with different aligners, such as STAR [71] and BWA [72]. Different aligners have their own advantages; for example, STAR software (version 2.7.6a), especially the “two-pass” mode, performs well at splicing junctions [71]. But optimizing the parameters, like mapping quality of a single aligner, might only slightly reduce the misalignments, while the alignments simultaneously supported by multiple aligners seem highly accurate. In fact, this strategy worked well when we identified A-to-I editing sites in the old genome assembly of honeybees.

It is intuitive to consider that a parameter controlling “how many mismatches are allowed” would affect the mapping accuracy. The edited reads contain additional mismatches compared to unedited reads, so the edited reads are less likely to be accurately aligned. However, the commonly used RNA-Seq aligners, like STAR, allow as many as N (N = 15% read length) mismatches in a single alignment [71]. A 150 bp single-ended read would allow 22 mismatches via STAR mapping. Although some studies aim to distinguish between “regular editing sites” and “hyper-editing sites” [7,66,73] based on number of mismatches per read, for common researchers using STAR [71], the alignment of most reads is not affected by whether reads are “edited or not”. Instead, some unknown intrinsic biases of each aligner that cause misalignments are inevitable so that one may consider only keeping the alignments supported by multiple aligners.

Before variant calling, there are still a few steps required to refine the alignments. For example, normal transcriptome analyses other than transposons studies usually require only keeping uniquely mapped reads [3,4,6,7], which means that the reads mapped to multiple genomic loci are not considered. Then, PCR duplicates should be removed by well-established tools (https://broadinstitute.github.io/picard/) (accessed on 2 June 2022). Since these filtering steps are commonly used in transcriptome studies that are not necessarily specific to RNA editing, we will not highlight the detailed procedures and pipelines.

## 6. Variant Calling: Which Reads and Which Bases Should Be Used?

When mapping, one presumes that the reads were accurately sequenced so as to determine which genomic position the reads came from. As described above, the unreliable alignments are removed from the downstream analyses. But during calling variants, one should be aware that there might be sequencing errors in the reads, so those error bases must be excluded to find the real RDD. For commonly used variant callers like samtools [74] and GATK [75], the bases with low sequencing quality could be discarded with “-Q M”. When M = 20, the bases with <99% accuracy are discarded; when M = 30, bases with <99.9% accuracy are removed. Note that the filter on base quality will help the variant calling only when the alignment is accurate. As we have stated, undesired mismatches mainly come from misalignments. If a read is mis-aligned, even if one only maintains the 100% accurate bases, one will also inevitably find false-positive mismatches which do not reflect the real RDD.

Moreover, in many cases, the mismatches at both ends of the reads (5–6 bp) are discarded since read ends tend to have higher sequencing error rate reflected by lower sequencing quality. This strategy worked well in many studies [4,68] where researchers observed that mismatches were enriched in read ends [76]. Again, trimming both ends of the reads is useful only when the alignment is accurate. If the read is misaligned, then the mismatches could appear in any position rather than at both ends. We aim to introduce two additional issues related to mismatches and read ends. (1) Soft-clipping: This terminology describes the reads which have partially mapped to a region but another part is unaligned, like the case shown in Figure 3C. While soft-clipped alignments might contain some misalignments, it should be stressed that many of the soft-clipped reads in RNA-Seq are accurately aligned: a read from mature mRNA that spans splicing junctions will be split into two parts when aligned to the genome (Figure 3D), then the read can only be computationally labeled as “soft-clipping” (symbol S), but the mapped locus is actually correct. Traditionally, soft-clipped parts in the reads were not considered by the variant calling tools. Accordingly, considering the tendency of soft-clipping near splicing junctions, the variants near splicing sites were discarded. (2) The “ReadPosRankSum” parameter in GATK: The capability of GATK software (version 4.3.0.0) is reflected in many aspects. An example is the “ReadPosRankSum” parameter [75]. For each variation site, this parameter tells us whether the bases supporting the reference allele and bases supporting the alternative allele have a preference in their positions on reads. For instance, if the bases supporting the reference allele are smoothly distributed along different reads while the bases supporting the alternative allele are enriched in reads ends, then this is a strong warning that the variation might come from sequencing errors. The hard filter of GATK would consider this issue. Some broadly used editing detection tools like HPC-REDItools [77] enable the control for base quality and mapping quality and support the removal of read ends. The commonly considered filters and criteria about variant calling could be achieved by REDItools. But since its input file is the sequence alignment Bam file, the mapping step can’t be controlled by this tool. It is still up to the users to carefully ensure the accuracy of the provided alignment file.

**Figure 4:**
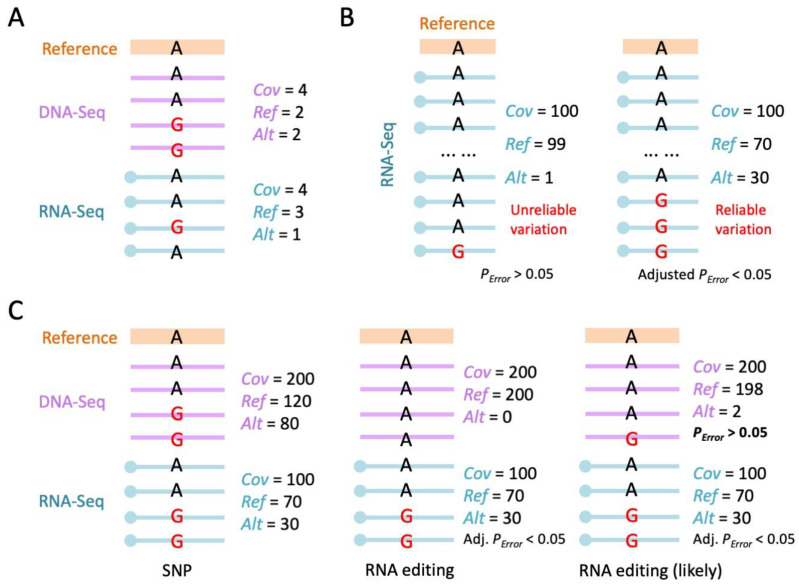
Signatures of reliable RNA editing sites. (**A**) Definition of *Ref*, *Alt*, and *Cov* counts in DNA-Seq and RNA-Seq data. Here, the reference allele is A, and the alternative allele is G. (**B**) Examples of unreliable and reliable variations in RNA-Seq based on *Ref*, *Alt*, and *Cov* counts. One alternative allele out of one hundred covered reads is unreliable and likely caused by sequencing error. (**C**) Identification of real RDD by RNA-Seq coupled with DNA-Seq. If the DNA-Seq reads show no signal of SNPs while RNA-Seq reads show reliable variation, then this site is likely to be an RNA editing site.

Next, after successfully determining which reads and which bases to be used for variant calling, most software will involve a “pipe-up” strategy and produce similar results (Figure 4A). The pipe-up step reveals the numbers of reads supporting the reference allele (*Ref*) and the alternative allele(s) (*Alt*) at each genomic position. The sequencing coverage on each site is *Cov* = *Ref* + *Alt* (Figure 4A). The identification of RNA editing sites usually requires the following steps that need to be specified.

In RNA-Seq data, if a variation site has *Cov* = 100 and *Alt* = 1, then this alternative base might come from sequencing error because although the base quality Q has already been controlled, sequencing errors still exist. In contrast, if a variation site has *Cov* = 100 and *Alt* = 30, then this site is likely to be a real variation between the RNA reads and the reference genome (Figure 4B), rather than sequencing error produced after library construction. The probability of an observed variation coming from sequencing error could be judged by a simple binomial test based on *Cov* and *Alt* numbers; the formula is *P_Error_* = pbinom (*Alt*-1, *Cov*, prob = eps0, lower.tail = F), where eps0 is the sequencing error rate which is approximately 0.1% in next-generation sequencing [7,63]. When *Cov* is fixed, *P_Error_* decreases with *Alt*. If *P_Error_* < 0.05 after multiple testing correction [78], it means that the variation observed is unlikely due to sequencing error and should be regarded as a genuine difference between the RNA reads and the reference genome.

The variations in RNA-Seq against the reference genome do not certainly represent the RDD because the SNPs will produce identical observations between RNA and the reference genome (as we illustrate in Figure 3B). To identify RNA editing sites, we should ensure that the matched positions in the DNA-Seq show clean signals of a “pure reference allele” (Figure 4C, middle). For example, for a site with RNA-Seq *Cov* = 100 and *Alt* = 30, if the DNA-Seq shows *Cov* = 200 and *Alt* = 80, then this site is likely to be a heterozygous SNP (Figure 4C, left). In contrast, if this site has DNA-Seq *Cov* = 200 that all support the reference allele, then the variation in RNA-Seq should be real RDD explained by RNA editing (Figure 4C, middle). Thus, it seems that one could simply use a criterion of “DNA *Cov* > 0 and *Alt* = 0” to ensure the “purity” of DNA-Seq. This is also the criterion used by studies from prestigious groups [7]. Notably, a super-meticulous method would consider that DNA-Seq is also subjected to sequencing errors. If a site has DNA *Cov* = 200 and *Alt* = 2, then this 1% variation level does not justify an SNP, and the two alternative bases are probably sequencing errors. The solution is to perform a similar binomial test on DNA *Cov* and *Alt*. Sites with *Cov* > 0 and *P_Error_* > 0.05 meaning that the alternative bases in DNA (e.g., 2 out of 200 bases) might come from sequencing errors so that there is actually no DNA polymorphism at this position (Figure 4C, right).

Taken together, after obtaining the reference and alternative allele counts for both RNA and DNA, the most meticulous criteria for a reliable RNA editing site are RNA-Seq *P_Error_* (adjusted) < 0.05 and DNA-Seq *Cov* > 0 and *P_Error_* > 0.05. Nevertheless, in some highly acknowledged studies, the criteria for DNA-Seq are simplified as *Cov* > N and *Alt* = 0 [7,63].

## 7. Hyper-Editing Pipeline Retrieves the Unmapped Reads

**Figure 5:**
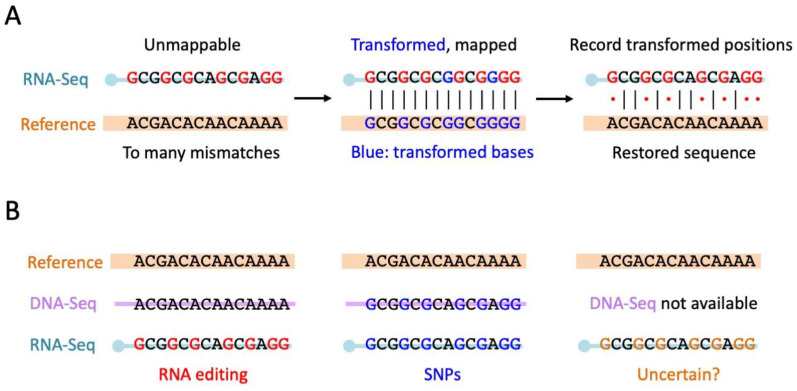
Hyper-editing pipeline. (**A**) The principles of the hyper-editing pipeline. The heavily edited reads cannot be mapped to the genome. An A-to-G transform on both reference genome and sequencing reads will solve this problem. (**B**) Limitations of the hyper-editing pipeline if a matched DNA-Seq is not available. The RNA editing sites identified might be clustered A-to-G SNPs.

In addition to the regular RNA editing sites identified by traditional variant calling pipeline on RNA-Seq and DNA-Seq data, the hyper-editing pipeline [66,79] tries to identify the extensively edited RNAs. Hyper-editing sites have no strict definition; they are usually highly clustered and located in lowly expressed regions. Although the regions have low sequencing coverage, the covered transcripts are all heavily edited [79]. As the original study claimed, “hyper-edited regions typically do not express unedited transcripts” [79]. Thus, hyper-editing sites are not measured by editing levels and instead the “number of hyper-edited reads” or “number of editing events per read” should be more informative. The initial trigger of this hyper-editing strategy is some heavily edited reads (RNA-Seq) failing to be mapped to the reference genome due to too many A-to-G mismatches (Figure 5A). The entire hyper-editing pipeline aims to rescue these unmapped reads. Notably, we revealed that STAR software (version 2.7.6a) [71] enables accurate alignments with numerous mismatches, but here we will not thoroughly discuss which aligner should be used for the hyper-editing pipeline. The group of its creator has chosen BWA to map the reads, and this workflow has already been well established and highly acknowledged [66,79].

The hyper-editing pipeline deliberately collects the unmapped RNA reads and aims to determine which of these reads are unmapped due to excessive A-to-I RNA editing [66]. The problem is how to distinguish the highly edited reads from those “truly unmapped” reads (e.g., reads from contamination). To resolve this issue, the hyper-editing pipeline transforms all adenosines to guanosines for both the unmapped reads and the reference genome [66]. After this A-to-G transformation, no matter how extensively a read has been edited, it should be mapped to the transformed reference genome (Figure 5A). In contrast, the contamination reads cannot be mapped to the genome, even with the A-to-G transformation. In this way, the hyper-edited reads can be located to the genome for further annotation. Then, the original adenosines at the A-to-G transformed positions are restored for both RNA-Seq and reference genome, and then the mismatches between the original RNA sequence and the genome can be explicitly demonstrated (Figure 5A). This transformation process is repeated for other types of mismatches, and then all the types of variations are recorded if available. Not surprisingly, the final profile usually shows that the majority of the mismatches are A-to-G [66], suggesting prevalent hyper-editing events. This is expected because, in theory, other types of mismatches usually come from unfiltered SNPs or sequencing errors and should not exhibit a tendency to “cluster the same type of mismatch within the same read”. A-to-I hyper-editing typically takes place in the repetitive regions targeted by mammalian ADAR1. Model insects *Drosophila melanogaster* and *Apis mellifera* do not have such abundant repeats like humans, and thus the hyper-editing in flies and bees might be less abundant. For RNA editing in cephalopods, as the transcript sequences were used as references, the CDS editing sites rather than repetitive editing sites were investigated as a priority [7,32].

Overall, the hyper-editing pipeline with the transformation strategy is a very well-established pipeline. Readers may commonly envision an extreme situation and raise a potential concern: “if every adenosine is edited, is it possible to identify the editing sites via that hyper-editing way?” (Figure 5A). The answer is yes. After transformation on both RNA reads and the reference genome, the sequences of RNA reads would be completely identical to the reference genome (Figure 5A). There is no reason why the RNA reads could not be mapped to the reference genome. Once a read is mapped, then its genomic location, together with the locations of all “converted sites” in that read, is known. Thus, all the information needed for an editing site is obtained. This logic flow is proposed in the original study [66]. There is no need to determine the editing sites in the highly edited reads.

As we have mentioned, the RNA aligner STAR [71] allows as many as 15% mismatches of the reads, implying that even a 150 bp read has 22 A-to-G mismatches due to RNA editing, and it could still be mapped to the reference genome. However, the hyper-editing methodology [66] was proposed only shortly after the appearance of STAR [71], so the pipeline used an earlier published aligner BWA [72]. Moreover, since the hyper-editing pipeline is a DNA-free method that does not require DNA-Seq data from matched individuals, it might misidentify some false-positive variations derived from neighboring A-to-G SNPs (Figure 5B). Nevertheless, we argue that the case of clustered A-to-G SNPs should be very rare, and the hyper-editing pipeline itself does not prevent us from using DNA-Seq data to improve the accuracy of RNA editing sites. A hyper-editing analysis with a matched DNA-Seq from the same individual is highly recommended. Then the false-positive sites with clustered SNPs are excluded. Indeed, mapping a DNA read full of A-to-G SNPs to the reference genome cannot be accomplished by normal mapping tools like BWA, so that the mapping process might again entail the transform strategy.

Normally, the use of DNA-Seq data to facilitate hyper-editing detection would successfully remove some scattered SNPs in a series of hyper-editing sites. For example, if the hyper-editing pipeline identifies 10 RNA editing events in a 150bp read of RNA-Seq, but after mapping the DNA-Seq data to the reference genome, it is found that one of these ten positions is actually an A-to-G SNP, then the cluster of hyper-editing events would be corrected to nine editing events. Therefore, the normal DNA-mapping strategy is enough to meet the requirements for SNP calling and then correcting the hyper-editing results.

## 8. Experimental Verification of RNA Editing Sites

All genome-wide in silico analyses need to be partially verified to become more convincing and widely accepted. For A-to-I RNA editing events, Sanger sequencing on both RNA and the matched DNA sequences is the best verification. The editing level could also be read from the Sanger traces.

**Figure 6:**
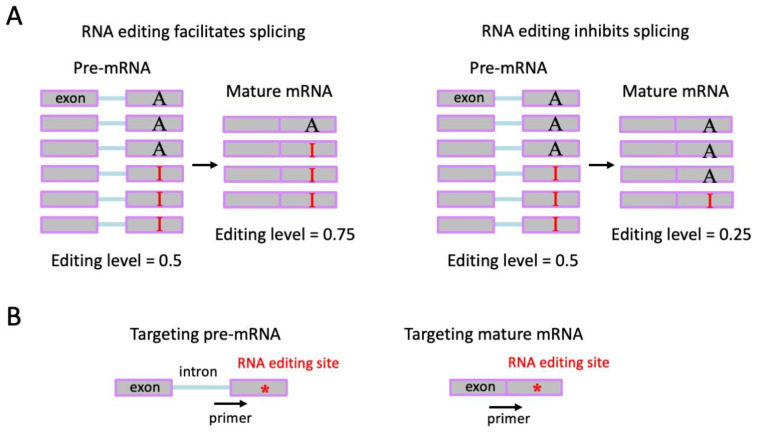
Sanger verification of A-to-I RNA editing sites and the potential pitfalls and guidance. Sanger sequencing of the RNA and DNA from the match individual is the best verification of RNA editing events. (**A**) Why and how the editing levels in pre-mRNA and mature mRNA could be different. If the RNA editing event affects splicing efficiency, then the editing levels will be different between pre- and mature mRNAs. (**B**) Sequencing the editing site (represented by “*”) in pre-mRNA and mature mRNA requires different designs of primers. The purpose of this step is to check whether the editing levels are different between pre- and mature mRNAs.

One should be aware that the editing level in NGS is a mixture of pre-mRNA and mature mRNA (Figure 6A). For Sanger sequencing, primers should be designed to fit either the pre-mRNA sequence or the mature mRNA sequence or both (Figure 6B). Editing levels in pre-mRNA and mature mRNA might be different. There are plenty of reports suggesting that A-to-I RNA editing and alternative splicing affect each other [80,81,82,83] as both processes occur in the nucleus. If an editing event increases splicing efficiency, then the edited pre-mRNAs would contribute more to the mature RNA pool compared to the unedited pre-mRNAs, leading to a higher editing level in mature mRNA than in pre-mRNA (Figure 6A), and vice versa. It should be noted that this difference in editing level could only be detected by Sanger sequencing with distinct primers. In NGS short reads, the editing level is averaged for pre-mRNA and mature mRNA.

## 9. In Silico Verification of RNA Editing Sites

The Sanger verification of the A-to-I RNA editing site is not always available for the following reasons: (1) Some samples like small-sized insects were rare, and no specimen was left after constructing the NGS libraries; (2) Even there is specimen left at this stage, it is not a living sample. All specimens are fast-frozen during sample collection. RNAs might be degraded after such a long period. (3) Many comparative genomic studies on the evolutionary landscape of RNA editing use public data and do not have the matched samples at all [35]. For these reasons, in silico verification of RNA editing sites is required.

**Figure 7:**
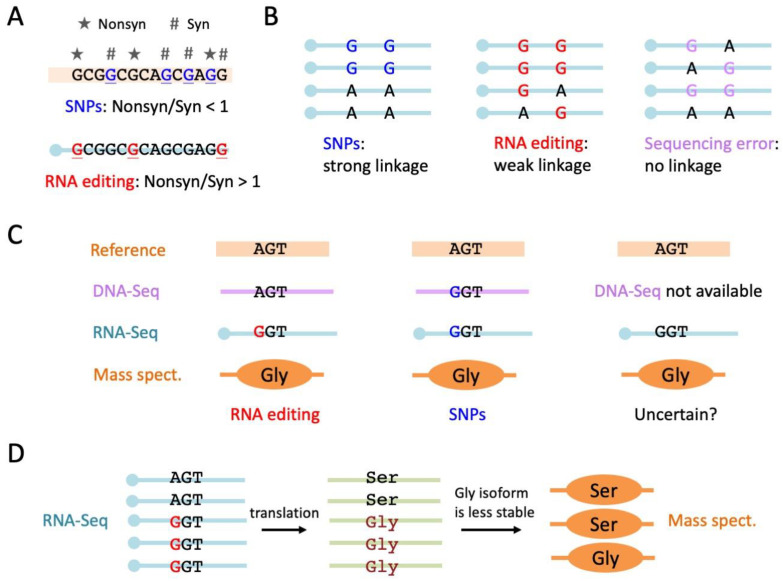
Several ideas for in silico verification of A-to-I RNA editing sites. (**A**) Nonsyn/Syn ratio of RNA editing sites or SNPs. SNPs or RNA editing sites are underlined. Overrepresentation of Nonsyn editing sites is a signal of positive selection, negating the possibility of the variations coming from artefacts. (**B**) Linkage disequilibrium (LD) between SNPs (strong linkage), RNA editing sites (weak linkage), and sequencing errors (no linkage). (**C**) The mass spectrum (MS) is not a verification of RNA editing if DNA-Seq is not available because SNPs could not be excluded. (**D**) The use of the mass spectrum to study the effect of nonsynonymous editing sites. If the pre-edit and post-edit protein isoforms have differential stability, then one should observe differential “editing levels” in RNA-Seq and MS data.

The first method of in silico verification is to calculate the nonsynonymous to synonymous ratio of editing sites, denoted as Nonsyn/Syn [84] (Figure 7A). SNPs are one of the major confounding factors that hamper the accurate identification of RNA editing. Most nonsynonymous SNPs are deleterious and should be eliminated by purifying selection. Among the extant SNPs, nonsynonymous mutations are largely depleted, and the Nonsyn/Syn ratio is much lower than 1. In contrast, the insect Nonsyn RNA editing sites seem to be positively selected due to their ability to flexibly diversify the proteome. The Nonsyn/Syn ratio of RNA editing sites should remarkably exceed the random expectation [84]. By examining the Nonsyn/Syn ratio of the identified RDDs, one can obtain a rough estimation of whether these RDDs are authentic RNA editing sites or they still contain many false-positive variations. Indeed, this methodology only serves as a confirmation when the RNA editing sites are accurately identified, but when the RDDs are filled with false-positive sites, this method does not help refine the results.

Another in silico verification method is to check the linkage between RNA editing sites (Figure 7B). Among the numerous reads in the RNA-Seq data, the variation sites against the reference genome might contain RNA editing sites, SNPs, and a few sequencing errors. These three groups of variations should have the following distinguishable patterns: (1) RNA editing sites are highly clustered in the genome but are weakly linked in the reads; (2) SNPs do not show a strong cluster in genome distribution but should be strongly linked in the reads; (3) sequencing errors do not show a cluster or linkage at all (Figure 7B). The reasons for these patterns are clear. SNPs detected in the RNA-Seq all come from the same genome, so they would show strong linkage in the transcripts as well. RNA editing events in the transcriptome take place co-/post-transcriptionally, so they show independence to some extent. However, editing events are not completely independent due to the “batch production” property of editing enzyme Adar [79]. This editing mechanism determines the linkage and cluster properties of RNA editing events in the transcripts. Nevertheless, it is conceivable that the strength of linkage between RNA editing events is weaker than the “complete linkage” between SNPs. A script for calculating the linkage disequilibrium (LD) between variations in RNA-Seq was previously developed to facilitate estimation of the reliability of RNA editing sites. Based on the LD and cluster features of RNA editing sites, two DNA-free methodologies termed GIREMI [85] and SPRINT [73] were invented to identify RNA editing sites in the transcriptome data. Again, we stress that these bioinformatic methodologies [73,85] are only suitable for the genome-wide profiling of the RNA editome, and they have less power in determining whether a single site is a high-confidence RNA editing site.

Note that for Nonsyn editing sites, using mass spectrum (MS) data is not a verification [86] (Figure 7C). The MS can only verify the difference between RNA and the reference genome; it is not an indication of the RNA–DNA difference because the DNA resequencing is not tested by the MS. The change in protein sequence could still come from a SNP (Figure 7C). When the authenticity of editing sites has been proved by previous steps, then the MS data could have other usages in studying the effect of nonsynonymous editing sites [7]. For example, the previous study in cephalopods found that editing levels in the MS increased with the levels in NGS [7]. This positive correlation, although expected, should confirm that there are no negative feedback mechanisms to suppress the translation of edited mRNAs compared to unedited mRNAs. Notably, we stress that since the MS is a semi-quantitative method, the MS data on the entire genetic product do not exist, and they are likely to be available only for a portion of the products. It seems that the MS should not be used for verifying genome-wide nonsynonymous editing. Like using Sanger sequencing to verify a small fraction of editing sites, the MS data could be used to verify the few nonsynonymous editing sites covered by the peptide where the DNA sequence has already been confirmed [7]. For another example of the usage of MS data, presume that if the edited version produces a less functional protein isoform, then we should expect a lower “editing level” in the MS compared to RNA-Seq because the edited protein isoform is more likely to be degraded (Figure 7D). This prediction could be systematically verified by the joint analysis of multi-omics data, and again only the sites covered by the peptides could be studied. Nevertheless, as we have emphasized, MS data are not an indication of the authenticity of editing sites. Presume that this position is a heterozygous SNP, then the relative fractions of the two alleles might also differ between RNA-Seq and the MS due to differential protein stability of the two isoforms (Figure 7D). Taken together, MS data should be cautiously used in the study of RNA editing.

In addition to the aforementioned in silico methods, further supporting evidence for the reliability of RNA editing sites is the enrichment in dsRNA structure. Bioinformatic tools like RNAfold and RNALfold were used to predict RNA secondary structure from the primary sequence [87]. Transcriptome-wide analyses in *Drosophila* and honeybees revealed that the edited adenosines had significantly larger fractions in dsRNA compared to unedited adenosines. However, this difference was not a 100% versus 0% contrast as editing sites usually have exceptions that are located outside dsRNA. We can only state that the global RNA editing sites are reliable as they exhibit enrichment in dsRNA, but for an individual editing site the reliability cannot be judged even it is located in the dsRNA structure. Researchers have found that dsRNA is an important element affecting editing efficiency [36,37] but is not the only determinant of the presence or absence of editing.

## 10. Future Directions

In this review, we first introduced the occurrence, regulation, and importance of A-to-I RNA editing in metazoans and then used insects as an example to describe the procedures and difficulties in the accurate identification of RNA editing sites from the transcriptomes. Meanwhile, we provided future directions that seem promising in this field. In summary, (1) to obtain an accurate genome-wide profile of RNA editing sites, a transcriptome coupled with the DNA resequencing of a matched sample is favorable; (2) currently, the single-cell RNA sequencing technique is not well applied to RNA editing studies, but this is a promising aspect and future direction that many researchers are making efforts toward; (3) during mapping and variant calling steps in bioinformatic analyses, various issues like mapping quality, base quality, soft-clipping, splicing junctions, and the positions of nucleotides on a read should be carefully considered; (4) low-throughput Sanger sequencing is the best for the verification of RNA editing sites, but the nonsynonymous-to-synonymous ratio and the linkage information serve as auxiliary evidence for the reliability of RNA editing sites.

We believe that bringing these thoughts to the readers will help them clarify future directions. Researchers might (1) obtain a clearer picture on the landscape of RNA editomes in metazoan species; (2) understand the advantages and limitations of current methodologies used in RNA editing identification and how to improve them; (3) choose the currently most appropriate methodology and experimental design to fit their own work; (4) increase the accuracy of RNA editing detection and thus benefit the development of the whole field; (5) avoid futile efforts in investigating RNA editing with unsuitable datasets; and (6) promote the development of new methodologies in either experiments or bioinformatics that could overcome the current limitations.

## 11. Conclusions

We have systematically reviewed and summarized the knowledges in the significance of RNA editing, highlighted the promising aspects of the development of this field, and meanwhile raised several challenges in the accurate identification of such editing events. With insightful perspectives on both scientific and technical details, our review will benefit the researchers in the broader RNA editing community.

## Abbreviations

ADAR	adenosine deaminase acting on RNA
A-to-I	adenosine-to-inosine
dsRNA	double-stranded RNA
LD	linkage disequilibrium
MS	mass spectrum
NGS	next generation sequencing
RDD	RNA-DNA difference
scRNA-Seq	single cell RNA-sequencing
SNP	single nucleotide polymorphism
TCGA	The Cancer Genome Atlas
